# Evaluation of Mercury Contamination in Chickens (*Gallus gallus*) and Soils in an Artisanal Gold Mining Area in San Martin De Loba, Bolivar, Colombia

**DOI:** 10.1007/s00128-025-04096-9

**Published:** 2025-09-09

**Authors:** Juliana Ramirez-Ortiz, Margareth Duran-Izquierdo, Lucellys Sierra-Marquez, Jesus Olivero-Verbel

**Affiliations:** Environmental and Computational Chemistry Group, School of Pharmaceutical Sciences, University of Cartagena, Zaragocilla Campus, Cartagena, 130015 Colombia

**Keywords:** Birds, Feathers, Poultry, Bioaccumulation, Arjona

## Abstract

The use of mercury (Hg) in artisanal gold mining in San Martin de Loba (SML), Bolivar, Colombia, poses significant environmental and health risks. This study aimed to evaluate total mercury (T-Hg) concentrations in chicken feathers (*Gallus gallus*) and soils from SML, and compare them with those obtained in a reference site without mining activity (Arjona). A total of 40 chickens and 30 soil samples were taken in SML, along with 31 chickens and 21 soil samples in Arjona. Using the Lumex RA-915 + analyzer, mean T-Hg levels in breast feathers, wing feather rachises, and barbs were 2.37 ± 0.42, 0.72 ± 0.32, 2.97 ± 1.26 µg/g in SML, and 0.41 ± 0.05, 0.23 ± 0.04, 0.76 ± 0.07 µg/g in Arjona, respectively. The average total mercury (T-Hg) concentration in soils from SML was 45.5 ± 12.4 µg/g, markedly exceeding the levels found in Arjona (0.04 ± 0.001 µg/g). The findings indicate severe contamination in SML soils and bioaccumulation in local birds eaten by humans, representing health risks to consumers.

## Introduction

Environmental pollution is one of the primary challenges for humanity and currently contributes to a significant number of diseases globally, particularly in low- and middle-income countries (Landrigan and Fuller 2014). While pollution encompasses both organic and inorganic substances, the concern over heavy metals is particularly pronounced due to their toxic properties (Tchounwou et al. 2012), with mercury (Hg) standing out as one of the most impactful pollutants worldwide, especially in Latin America (Canham et al. 2021), where its high persistence, bioaccumulation, and biomagnification in biota through the trophic chain exacerbate its environmental and health impacts (Buch et al. 2017). In Colombia, artisanal gold mining is a major contributor to Hg pollution (Palacios-Torres et al. 2018), making the country the second-largest emitter of Hg in air and water globally, and the highest per capita, with approximately 2.261 mining titles and 6.330 extraction points (de Paula-Gutierrez and Agudelo 2020). Metallic mercury (Hg^0^) is used to amalgamate gold, and once the amalgam is burned, it releases Hg vapor into the atmosphere, which is then deposited in ecosystems (Crespo-Lopez et al. 2021), contributing to Hg-rich tailing formations at mining sites (Olivero-Verbel et al. 2016).

Gold mining in Bolivar represents 4% of Colombia’s national gold production. San Martin de Loba (SML), in southern Bolivar, is marked by underground and alluvial mining, with artisanal operations being common. Nearby communities face Hg exposure through contaminated fish and possibly meat, as livestock and birds ingest polluted food and water (Fernandes et al. 2020; Zhuang et al. 2014). Free-ranging poultry scavenge around mine tailings, increasing their Hg exposure (Chibunda and Janssen 2009). Despite these risks, few studies focus on domestic animals in mining areas. Feathers, widely used as bioindicators of contaminant exposure (Gonzalez et al. 2018; Thakur et al. 2020), can contain up to 70% of a bird’s total Hg load (Ochoa-Acuña et al. 2002; Sierra-Marquez et al. 2018). Conversion ratios like 7:1 or 10:1 (feather: muscle) allow non-lethal estimation of Hg in tissues (Thompson et al. 1990), helping assess human health risks from avian consumption (Burger and Gochfeld 1997). This study analyzed T-Hg in chicken feathers and soil from SML, comparing them to a non-mining site to evaluate contamination levels.

## Materials and Methods

### Study Area

**Fig. 1:**
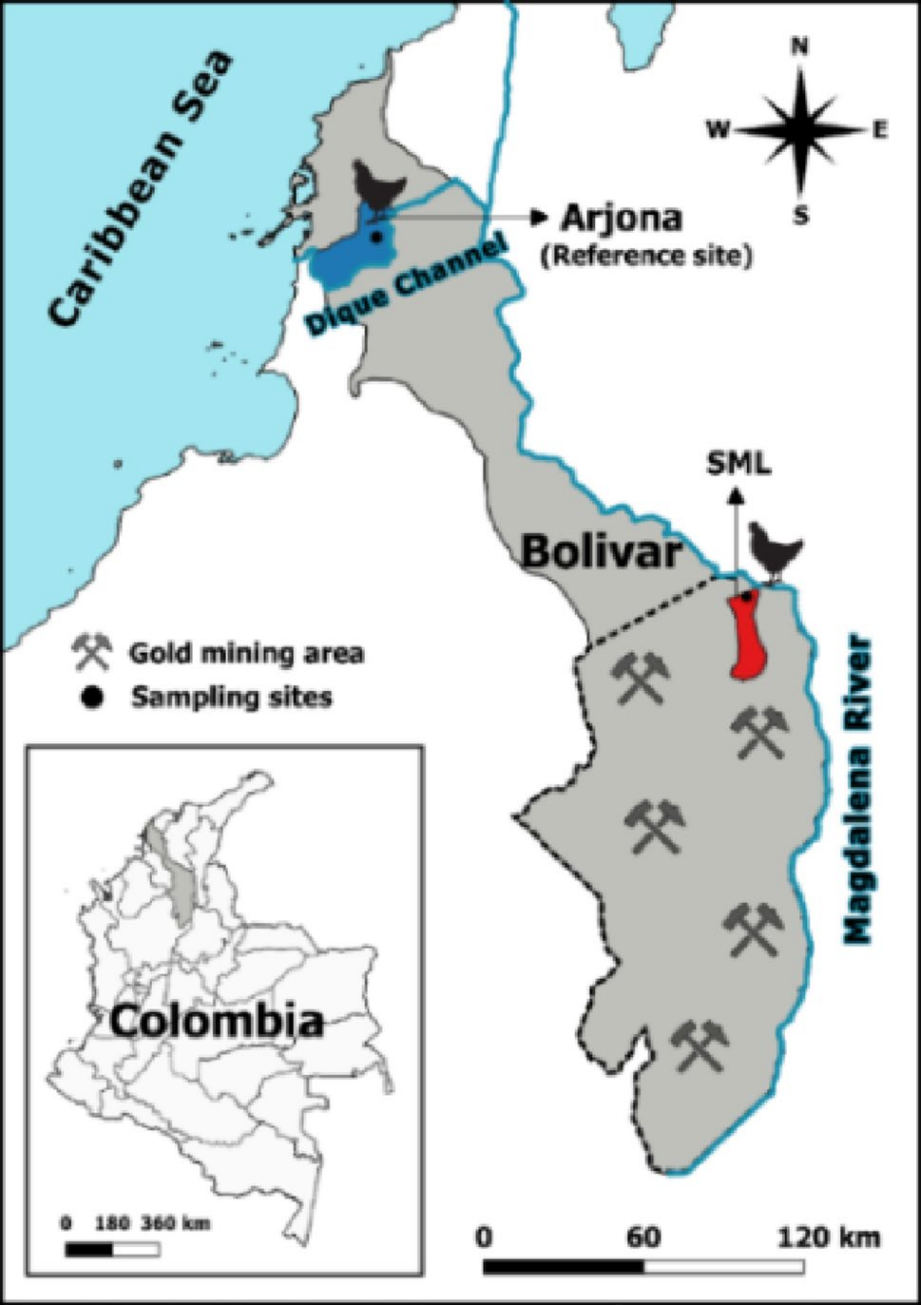
Location of sampling sites in the department of Bolivar, Colombia

The SML Mining District is located in southern Bolivar, an alluvial plain between the Magdalena and Cauca rivers, with swamps and high temperatures throughout the year (Olivero-Verbel et al. 2014). The reference site for this study was Gambote, a township next to the Dique Channel, a distributary of the Magdalena River. While gold mining is the main economic activity in the mining district, fishing and agriculture are also common in both regions. Sampling points were selected based on the presence of the target species (*G. gallus*) and are illustrated in Fig. 1.

### Collection and Treatment of Samples

Chickens were randomly selected from an area of active gold-mining operations in the SML region. These free-roaming birds were captured, weighed, and breast and wing feathers were collected and sealed in pre-labeled bags. Surface soil samples were collected in small plastic containers, combining three subsamples taken at a depth of 0–10 cm from the surface. The feathers were washed sequentially with detergent, acetone, and deionized water to remove external contaminants (Keute et al. 2024), dried at 50 °C for 6–12 h, then cut into 2–3 mm pieces. Wing feathers were separated into rachis and barbs. For breast feathers, however, this division was not performed due to the thinner rachis. Soil samples were lyophilized using a freeze dryer (Labconco FreeZone 2.5 Plus) for 24–48 h, then homogenized and sieved to obtain particles smaller than 1 mm.

### Determination of Total Mercury (T-Hg)

T-Hg concentrations (dry weight) were quantified using a direct mercury analyzer (Lumex RA-915 + with PYRO-915 + solid module) on 3–5 mg of feather and soil samples. Calibration curves, constructed with at least five points using Certified Reference Materials (CRMs) from the National Research Council of Canada, were considered suitable with a regression coefficient of ≥ 0.99. To ensure accuracy, IAEA-086 (0.573 µg/g T-Hg) and IAEA-085 (23.2 µg/g T-Hg) CRMs were used for feather samples, and IAEA-158 (0.132 µg/g T-Hg) for soil samples. Precision was verified with blanks and CRMs, yielding recovery rates of 98.4% for feathers and 96.9% for soils. Each sample was analyzed in duplicate, with a coefficient of variation of less than 15% between replicates, and limits of detection (LOD) were 0.004 µg/g for feathers and 0.005 µg/g for soils, while limits of quantification (LOQ) were 0.011 µg/g and 0.012 µg/g, respectively.

### Determination of Soil Contamination

Soil contamination with Hg was evaluated using the geoaccumulation index (I_geo_) (Müller 1981), using 0.06 µg/g as the geochemical background value of the metal (Berrow and Reaves 1984). Contamination levels were classified as follows: Class 0: I_geo_ ≤ 0 (unpolluted); Class 1: 0 ≤ I_geo_ ≤ 1 (from unpolluted to moderately polluted); Class 2: 1 ≤ I_geo_ ≤ 2 (moderately polluted); Class 3: 2 ≤ I_geo_ ≤ 3 (moderately polluted to strongly polluted); Class 4: 3 ≤ I_geo_ ≤ 4 (strongly polluted); Class 5: 4 ≤ I_geo_ ≤ 5 (strongly polluted to extremely polluted); Class 6: I_geo_ >5 (extremely polluted).

### Estimation of Hg Levels in Poultry Muscle From Feathers

The T-Hg concentrations in the breast muscle of chickens were estimated using the mean T-Hg levels measured in breast feathers from birds in SML and Arjona, applying the suggested relationships (7:1 and 10:1) and the quadratic equation proposed by Kim et al. (2019).

### Data Analysis

Results are presented as mean ± standard error (µg/g or ppm). Due to non-normal distribution (Shapiro-Wilk test) and unequal variance (Bartlett’s test), the Mann-Whitney U test compared T-Hg concentrations between sites. Differences among feather parts were assessed using the Friedman test with Dunn’s post hoc test. Spearman’s correlation analyzed variable associations. Statistical analyses were performed in GraphPad Prism 5.01 (*p* < 0.05).

## Results

### Total Mercury (T-Hg) Concentrations

**Fig. 2:**
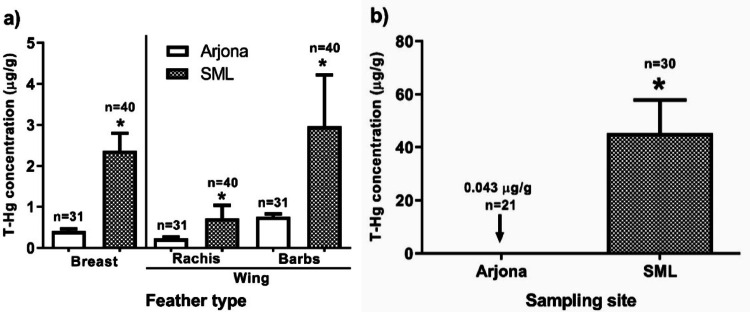
Mean T-Hg concentrations (µg/g, dry weight) obtained from **a** *G. gallus* feathers (Arjona, *n* = 31; SML, *n* = 40) and **b** soils (Arjona, *n* = 21; SML, *n* = 30) at the sampling sites. Error bars represent standard error of the mean

The mean T-Hg concentrations (dry weight) in all samples, including feathers and soils from the study sites, are presented in Table 1. In feathers, the values ranked from highest to lowest, were as follows: barbs (wing feathers) > breast feathers > rachis (wing feathers) at both sites (Fig. 2a). Statistical analysis revealed significant differences in T-Hg concentrations between all sample types from SML and Arjona, with higher values in the SML samples. Mann-Whitney U tests revealed statistically significant differences in breast feathers (U = 154.5, *p* < 0.0001) and soil samples (U = 0.0, *p* < 0.0001) (Fig. 2b), as well as in wing feather rachis (U = 350.5, *p* = 0.0018) and barbs (U = 353.5, *p* = 0.0020), confirming consistently higher T-Hg concentrations in SML across all sample types.

**Table 1:** T-Hg concentrations in feathers and soil from Arjona and SML

Sampling sites	Breast feathers (range)	Wing feathers (range)		Soil (range)
		Rachis	Barbs	
Arjona^a^	0.41 ± 0.05 (0.06–1.40)	0.23 ± 0.04 (0.07–1.21)	0.76 ± 0.07 (0.06–1.74)	0.04 ± 0.001 (0.007–0.08)
SML^b^	2.37 ± 0.42 (0.21–15.24)	0.72 ± 0.32 (0.04–12.85)	2.97 ± 1.26 (0.27–51.20)	45.5 ± 12.4 (1.58–245.05)

^a^Feathers, *n* = 31; soils, *n* = 21

^b^Feathers, *n* = 40; soils, *n* = 30. Mean ± standard error (µg/g, dry weight), ranges are shown in parentheses

In Arjona, significant differences were observed between breast feathers and the barbs of wing feathers, as well as between the rachis and barbs of wing feathers (Friedman test = 25.67, *p* < 0.0001). Meanwhile, in SML, T-Hg mean levels showed significant differences between breast feathers and the rachis of wing feathers, along with the two parts of the wing feathers (rachis and barbs) (Friedman test = 54.95, *p* < 0.0001) (Table 2).

**Table 2:** Comparison of T-Hg concentrations between Arjona (*n* = 31) and SML (*n* = 40) feathers

Samples	Arjona			SML		
	Friedman test	*p*-value	Dunn’s post test	Friedman test	*p*-value	Dunn’s post test
Breast feathers	25.67	< 0.0001	A	54.95	< 0.0001	A
Rachis (Wing feathers)			A			B
Barbs (Wing feathers)			B			A

Groups means sharing the same letter are not significantly different.

Spearman correlation analysis revealed a significant inverse relationship between T-Hg concentrations in the rachis and barbs of wing feathers and the weight of birds from Arjona, with moderate (ρ = − 0.565, *p* = 0.001) and weak (ρ = − 0.361, *p* = 0.046) correlation coefficients, respectively, while no significant associations were observed between T-Hg levels and weight in birds from SML.

Mercury levels varied according to feather type and parts, showing a significant association between T-Hg levels in the rachis and barbs of wing feathers in birds from both Arjona (*p* = 0.032) and SML (*p* < 0.0001), with a significantly stronger association in SML. Furthermore, at the latter site, T-Hg concentrations in breast feathers were also closely associated with those in the rachis and barbs of wing feathers (*p* < 0.0001) (Table 3).

**Table 3:** Spearman’s correlation between T-Hg concentrations of feathers

Samples	Arjona^a^		SML^b^	
	Breast feathers	Rachis (Wing feathers)	Breast feathers	Rachis (Wing feathers)
Rachis (Wing feathers)	0.111 (0.552)		0.585 (< 0.0001)*	
Barbs (Wing feathers)	0.351 (0.053)	0.386 (0.032)*	0.650 (< 0.0001)*	0.856 (0.0001)*

^a^*n* = 31

^b^*n* = 40. The *p*-value is shown in parentheses. *. Significant correlation, *p* < 0.05

### Estimation of Hg Concentrations in Muscle Tissue

The estimated T-Hg concentrations in chicken breast muscle from Arjona exceeded the European Union’s permissible limit for poultry muscle of 0.01 µg/g (European Commission 2018) by 5.9, 4.1, and 4.8 times, respectively, when using the 7:1 ratio, the 10:1 ratio, and the quadratic equation (Table 4). In SML, these values were even more concerning, surpassing the regulatory limit by 33.9, 23.7, and 16.8 times, respectively. All the estimated concentrations exceeded the provisional tolerable weekly intake (PTWI) for non-fish foods contaminated with Hg (0.004 µg/g), as established by the Codex Alimentarius (FAO/WHO 2019).

**Table 4:** Estimated T-Hg concentrations in breast muscle based on feather analysis

Sampling sites	T-Hg estimations in breast muscle (µg/g)		
	A	B	C
Arjona	0.059	0.041	0.048
SML	0.339	0.237	0.168

A: 7:1 ratio; B: 10:1 ratio; C: Quadratic equation proposed by Kim et al. (2019)

### Soil Hg Contamination

The soil Hg contamination assessment for both study sites indicated that all samples from Arjona were classified as unpolluted based on calculated I_geo_ (Table 5). In contrast, 33.3% of soils from SML were categorized as strongly to extremely polluted, and 66.6% as extremely polluted, with T-Hg concentrations reaching up to 245.05 µg/g in this mining area.

**Table 5:** Classification of the degree of contamination of soil samples with Hg at the sampling sites

Arjona^a^						
Group	*n*	Interval	Mean	I_geo_	I_geo_ Class	Classification
< 0.06	16	0.007–0.06	0.033 ± 0.00	− 1.45	0	Unpolluted
> 0.06 - <0.3	5	0.06–0.08	0.072 ± 0.00	− 0.32	0	Unpolluted
SML^b^						
> 0.3 - <5.0	10	1.58–4.64	2.74 ± 0.27	4.93	5	Strongly to extremely polluted
> 5 µg/g	20	5.88–245.05	66.82 ± 16.74	9.53	6	Extremely polluted

^a^*n* = 21; ^b^*n* = 30.

## Discussion

Diet is a key Hg exposure pathway in birds (Zhuang et al. 2014; Elsharawy 2015; Thakur et al. 2020), with their omnivorous habits indicating multiple sources, including invertebrates, plants, and contaminated soil. In SML, inhalation of inorganic Hg from mining amalgamation is a primary route. However, MeHg exposure cannot be excluded, as terrestrial birds also face MeHg contamination (Rimmer et al. 2010; Varian-Ramos et al. 2014), often via invertebrates like spiders that transfer MeHg from aquatic to terrestrial chains (Jackson et al. 2011) or through soil invertebrates under low pH and high organic matter (Nawrocka et al. 2020). The proximity of water bodies in SML may further facilitate MeHg transfer, increasing contamination in this mining-impacted area.

The elevated Hg concentrations observed in this study may pose risks to avian reproductive health, as Hg exposure is known to reduce reproductive success by decreasing the number of viable offspring (Whitney and Cristol 2018). Such effects are linked to eggshell malformations, teratogenicity, chick mortality, and abnormal behaviors, with Hg residues in feathers of 5–65 µg/g associated with these outcomes (Burger and Gochfeld 1997; Albuja et al. 2012), while levels as low as 0.5–11 µg/g are linked to reduced egg hatching and sterility (Eisler 2004). Although reproductive effects were not directly assessed in this study, mean T-Hg concentrations in feathers from birds in SML and Arjona exceeded the minimum threshold of this last range for potential reproductive toxicity. The unexpectedly high T-Hg levels in chickens from the reference site, Arjona, could stem from proximity to the Dique Channel, where significant Hg contamination in fish and sediments has been reported (Olivero et al. 1997; Olivero and Johnson 2002; Cogua et al. 2012), along with other potential sources such as locally grown grains irrigated with channel water or occasional ingestion of fish remains.

In other regions of Colombia, higher T-Hg levels in bird feathers have been reported compared to this study, with Gamboa-Garcia et al. (2019) documenting concentrations of 2.79–5.23 µg/g in *Pelecanus occidentalis* from the Pacific region, while Burgos-Nuñez et al. (2017) reporting similar values in seabirds from the Caribbean region, including *P. occidentalis* (5.15 ± 1.52 µg/g), *Phalacrocorax brasilianus* (4.99 ± 1.47 µg/g), *Fregata magnificens* (10.19 ± 4.99 µg/g), and *Thalasseus maximus* (3.57 ± 1.37 µg/g), likely reflecting the influence of the piscivorous diets of these species, which enhance Hg bioaccumulation. However, in Colombian gold-mining areas closer to our study site, Buelvas-Soto et al. (2022) reported lower T-Hg levels (0.47 ± 0.18 µg/g) in *Dendrocygna autumnalis* from the Mojana subregion, while Argumedo et al. (2013) documented 3.39 ± 0.63 µg/g in *G. gallus* feathers from the nearby Barranco de Loba municipality, Bolivar, exceeding concentrations observed in our study for all feather types and sections. Sierra-Marquez et al. (2018) found an average T-Hg concentration of 0.84 ± 0.05 µg/g in wild birds with diverse diets in Las Orquideas National Park in the Andean region, similar to the 0.72 ± 0.32 µg/g detected in wing feather rachises in our study but lower than the T-Hg concentrations in breast feathers and wing feather barbs (see Table 1).

The observed differences in T-Hg concentrations between wing feather sections (rachis and barbs) align with previous studies. Espin et al. (2012) reported higher T-Hg levels in wing feather barbs (2.66 ± 1.60 µg/g) compared to rachises (1.30 ± 0.76 µg/g) in *Alca torda* from the southwestern Mediterranean. Similarly, Gonzalez et al. (2018) found higher T-Hg concentrations in barbs than rachises for *Ardea alba* (0.62 > 0.36 µg/g) and *Egretta thula* (1.1 > 0.89 µg/g), although this pattern did not hold for *Nycticorax nycticorax* (0.34 < 0.45 µg/g) in Lake Chapala, Mexico. These variations may reflect tissue-specific deposition and accumulation (Calle et al. 2015) or differences in Hg affinity for feather proteins. In addition, the absence of significant correlations between T-Hg concentrations and bird weight in SML and Arjona suggests that feather bioaccumulation may be independent of body weight, potentially influenced by site-specific dietary and environmental exposure factors, given the free-ranging behavior of the birds. Nonetheless, further research is needed to better understand these relationships and the underlying mechanisms.

On the other hand, the high T-Hg levels detected in soil samples from SML, all surpassing the global average of 0.06 µg/g (Berrow and Reaves 1984), reflect the significant impact of mining activities in the region. These high concentrations are primarily attributed to the wet or dry deposition of atmospheric Hg (Hg^0^) released during amalgamation practices used in gold extraction. This contaminated soil subsequently acts as a reservoir of Hg, contributing to its transfer into biota and atmospheric and hydrological systems, aligning with global observations of exceptionally high Hg concentrations near sites in gold mining regions (Carranza-Lopez et al. 2019).

These T-Hg levels detected in SML soils surpass those reported for other artisanal gold-mining areas globally. In Indonesia’s Cikaniki River region, levels ranged from 0.11 to 7.0 µg/g (Tomiyasu et al. 2013), and later from 0.07 to 16.7 µg/g (Tomiyasu et al. 2017). In Brazil, studies documented lower concentrations, with 0.59 ± 0.003 µg/g in a mining site, 0.21 ± 0.005 µg/g in a deforested Amazon area, and 0.14 ± 0.005 µg/g in Risaralda, Colombia (Montoya et al. 2019). The values from this study also exceed those from the Magdalena Medio sub-region, Bolivar, where Carranza-Lopez et al. (2019) reported an average of 24.1 ± 9.4 µg/g, though their maximum value of 270 µg/g approximates the maximum observed here (245.05 µg/g). Additionally, Rocha-Roman et al. (2018) recorded a mean concentration of 3.4 ± 0.36 µg/g in SML, significantly lower than our findings, potentially due to sampling locations farther from mining activities, though their maximum value of 23.83 µg/g near mining sites aligns with the trends identified in this study.

### Limitations of Feather-Based Mercury Estimation

Conversion factors and predictive models for estimating Hg levels vary due to species differences, temporal changes, and environmental conditions. Research (Eagles-Smith et al. 2008; Low et al. 2020; Mukhtar et al. 2020) highlights inconsistencies tied to bird species, molting cycles, feather type, exposure pathways, and Hg speciation. Despite these challenges, this study underscores feather-based Hg analysis as a non-invasive, practical tool for estimating internal contamination.

This method helps assess Hg exposure through food chains. While non-fish sources pose lower risk due to shorter trophic chains, even low dietary concentrations (0.05 µg/g) can accumulate in bird tissues, posing health risks (Eisler 2004). Yin et al. (2017) found poultry near Hg mines in China exceeded intake limits, highlighting potential human health risks. In Hg-contaminated areas like SML, where poultry and other local foods are consumed, compounded exposure from air, water, and soil underscores the need for mitigation strategies to protect public health.

## Conclusions

This study expands mercury monitoring efforts by validating chicken feathers as effective, non-invasive bioindicators in mining regions. In the mining municipality of SML, located in southern Bolivar, chickens in gold mining areas exhibit significant T-Hg bioaccumulation in their feathers, representing a potential health risk for local populations, particularly those consuming poultry meat. While this risk exists in both SML and Arjona, it is markedly higher in SML due to elevated soil contamination linked to gold mining activities.
